# The dilemma of polypharmacy in psychosis: is it worth combining partial and full dopamine modulation?

**DOI:** 10.1097/YIC.0000000000000417

**Published:** 2022-07-12

**Authors:** Matteo Lippi, Giuseppe Fanelli, Chiara Fabbri, Diana De Ronchi, Alessandro Serretti

**Affiliations:** a Department of Biomedical and Neuromotor Sciences, University of Bologna, Bologna, Italy; b Department of Human Genetics, Radboud University Medical Center, Donders Institute for Brain, Cognition and Behaviour, Nijmegen, The Netherlands; c Social, Genetic & Developmental Psychiatry Centre, Institute of Psychiatry, Psychology & Neuroscience, King’s College London, London, UK

**Keywords:** antipsychotics, aripiprazole, dopamine antagonists, dopamine partial agonists, dopamine receptor, polypharmacy, psychosis

## Abstract

Antipsychotic polypharmacy in psychotic disorders is widespread despite international guidelines favoring monotherapy. Previous evidence indicates the utility of low-dose partial dopamine agonist (PDAs) add-ons to mitigate antipsychotic-induced metabolic adverse effects or hyperprolactinemia. However, clinicians are often concerned about using PDAs combined with high-potency, full dopaminergic antagonists (FDAs) due to the risk of psychosis relapse. We, therefore, conducted a literature review to find studies investigating the effects of combined treatment with PDAs (i.e. aripiprazole, cariprazine and brexpiprazole) and FDAs having a strong D_2_ receptor binding affinity. Twenty studies examining the combination aripiprazole – high-potency FDAs were included, while no study was available on combinations with cariprazine or brexpiprazole. Studies reporting clinical improvement suggested that this may require a relatively long time (~11 weeks), while studies that found symptom worsening observed this happening in a shorter timeframe (~3 weeks). Patients with longer illness duration who received add-on aripiprazole on ongoing FDA monotherapy may be at greater risk for symptomatologic worsening. Especially in these cases, close clinical monitoring is therefore recommended during the first few weeks of combined treatment. These indications may be beneficial to psychiatrists who consider using this treatment strategy. Well-powered randomized clinical trials are needed to derive more solid clinical recommendations.

## Introduction

Psychosis is a psychopathologic condition characterized by the presence of delusions and/or hallucinations, a loss/deficit of illness insight, and often it also involves changes in social interactions and cognition, as well as abnormal behaviors (Arciniegas, 2015; van Os and Reininghaus, 2016).

For many years, schizophrenia has been considered the archetype of psychosis (Arciniegas, 2015). However, psychosis can vary in intensity and frequency of symptom manifestations, as well as in impairment of daily functioning (Schrimpf *et al.*, 2018). For this reason, the existence of a symptomatologic continuum rather than isolated diagnostic categories for psychotic disorders has been theorized, leading to the definition of a schizophrenia spectrum, as also reported in the Diagnostic and Statistical Manual of Mental Disorders (DSM)-5 (American Psychiatric Association, 2013). However, psychotic symptoms may also appear in the context of other psychiatric disorders, including mood, substance use, and, in some cases, personality disorders, as well as in medical conditions, such as metabolic, neurologic, infectious, and inflammatory diseases (Lieberman and First, 2018).

The universally acknowledged pharmacologic treatment for psychotic disorders is based on antipsychotic medications, with all international guidelines specifically indicating antipsychotic monotherapy (APM) as the gold-standard treatment (Hasan *et al.*, 2012; NICE Clinical Guidelines, 2014; Galletly *et al.*, 2016; Remington *et al.*, 2017; Keepers *et al.*, 2020). Despite this, at least 20% of patients with schizophrenia do not have a sufficient response to APM (Asenjo Lobos *et al.*, 2010; Okhuijsen-Pfeifer *et al.*, 2018). Approximately 34% of patients with schizophrenia do not respond to the first- and second-line treatment with antipsychotics, and they are therefore classified as treatment-resistant schizophrenia (TRS) cases (Pae *et al.*, 2021). This is one of the reasons why antipsychotic polypharmacy (APP) is commonly prescribed in clinical practice (i.e. more than two antipsychotic drugs at the same time) (Lin, 2020). Some other motivations to choose APP over AMP include partial symptomatologic remission (e.g. the presence of residual depressive, cognitive, or negative symptoms), attempt of expediting treatment response, persistence of side effects of an effective APM (e.g. hyperprolactinemia or extrapyramidal symptoms) or clozapine intolerance and a severe course of the disease (Stahl, 2008).

According to a systematic review and a meta-regression that analyzed data up to the 2000s, the global average rate of APP was 19.6% in outpatients with schizophrenia (Gallego *et al.*, 2012). A recent cohort study, which studied a similar clinical population in Finland, indicated that 57.5% of patients received APP (Tiihonen *et al.*, 2019). A Turkish study reported APP in more than 70% of patients, with up to five simultaneous antipsychotics being prescribed (i.e. 44.4% of patients with two antipsychotics, 24.4% with three, 1.4% with four and 0.7% with five) (Yazici *et al.*, 2017). Therefore, APP is common, but with rates that are highly variable depending on the population considered.

A meta-analysis of randomized-controlled trials (RCTs) suggested that APP may be superior to APM in schizophrenia in terms of reduced treatment discontinuation and increased efficacy, particularly in patients with acute psychosis who were prescribed APP in an early phase combining two different antipsychotic classes [i.e. a first-generation (FGA) drug with clozapine or another second-generation antipsychotic (SGA)] for at least 10 weeks (Correll *et al.*, 2009). Interestingly, the same authors pointed out the lack of pharmacologic rationale for combining molecules with the same expected blocking potency on the dopamine receptor type 2 (D_2_), and the effectiveness of the combination did not appear as a linear function of antipsychotic dose and D_2_ blockade (Correll *et al.*, 2009; Correll and Gallego, 2012).

In 2014, a real-world study in patients with psychotic disorders (*N* = 14 150) observed that APM was superior to APP in relation to all-cause discontinuation, although APP outperformed APM in terms of reduced mortality and hospitalization rates (Katona *et al.*, 2014). This confirms that APP may be more appropriate during acute psychotic phases, when there is an increased risk of hospitalization and death, in line with the previously mentioned meta-analysis by (Correll *et al.*, 2009). The favorable effect of APP over APM on hospitalization rates was also confirmed by Tiihonen *et al.* (2019), who indicated a 7–13% lower risk compared to patients treated with APM. Noteworthy, clozapine plus aripiprazole (the first commercialized D_2_ partial agonist antipsychotic) was associated with a significantly better outcome in patients with schizophrenia compared to all AMP or APP alternatives and up to a 23% lower risk of psychiatric hospitalization compared to clozapine alone (Tiihonen *et al.*, 2019). Several other studies analyzed the combination of clozapine plus aripiprazole in patients with TRS (Porcelli *et al.*, 2012; Barber *et al.*, 2017). These studies report that aripiprazole add-on can be a useful, effective and tolerated strategy to improve residual or negative symptoms or reduce the risk of metabolic syndrome and weight gain. Similarly, other authors demonstrated the usefulness of combining aripiprazole with olanzapine to reduce weight gain and hyperlipidemia associated with the latter drug (Henderson *et al.*, 2009; Choi, 2015).

The use of aripiprazole in combination with another ongoing antipsychotic treatment is routinely considered for the treatment of antipsychotic-induced hyperprolactinemia. For example, risperidone, paliperidone, amisulpride, haloperidol and other FGAs are among the antipsychotics that are more likely to raise prolactin levels, and combining them with low doses of aripiprazole (i.e. 3– 6 mg/day) appears to be a well-tolerated and acceptable option (Domenico *et al.*, 2014; Taylor *et al.*, 2021). However, the benefit of combining higher doses of partial dopamine agonists (PDAs), such as aripiprazole, with high-affinity, full dopamine antagonists (FDAs) in case of partial response or side effects is less clear. In this regard, clinicians are often concerned about combining medications having distinct pharmacodynamic profiles in relation to dopamine receptor binding because of the risk of psychosis worsening or recrudescence (Stahl, 2012). The recent availability of new PDA (cariprazine and brexpiprazole) has made more relevant the need to investigate whether, in daily clinical practice, it is useful and well-tolerated to combine PDA and FDAs having a strong D_2_ binding affinity.

The aim of this review is to summarize the previous literature regarding the combined use of partial dopamine agonists with high D_2_ affinity and full dopamine antagonists in terms of potential clinical benefits and risks. Both studies in patients with psychotic disorders and animal models of psychosis were considered. Some key concepts related to the current classification of antipsychotics and their pharmacodynamic profile will be presented before addressing the main topic, as the pharmacodynamic profile of antipsychotics is generally considered the main ground to support the possible rationale of APP.

### Former classification of antipsychotics medications

The chemical structure of the available antipsychotics shows high variability, but they share the ability to block D_2_ to some extent (Li *et al.*, 2016). They are historically classified as FGAs (also defined as ‘typical’ antipsychotics or neuroleptics) and SGAs (also referred to as ‘atypical’ antipsychotics) (Stahl, 2021; Taylor *et al.*, 2021).

FGAs may be generally categorized into several chemical classes (e.g. phenothiazines and butyrophenones), and they are characterized by exerting their therapeutic effect by blocking D_2_ with various degrees of affinity without modulating serotonin receptors or the dopamine receptors type 3 (D_3_) or 4 (D_4_) (Stahl, 2021).

SGAs are distinguished from FGAs mainly for the combination of D_2_ antagonism or partial agonism with the block of serotonin receptor subtypes, such as the serotonin receptor 2A (5HT_2A_) and 2C (5HT_2C_). This dual action is the hallmark of SGAs pharmacodynamics and their ‘atypicality’. A higher antagonism on 5HT_2A_ is considered protective against the onset of iatrogenic extrapyramidal symptoms (Stahl, 2021).

Recently, research in psychopharmacology has led to the development of antipsychotics with a partial agonism on D_2_ (i.e., PDAs), such as aripiprazole, cariprazine and brexpiprazole. PDAs are sometimes referred as ‘third-generation antipsychotics’ (Mailman and Murthy, 2010) but they are in general considered as atypical antipsychotics for their antagonism on 5HT_2A_. These compounds act as stabilizers of dopaminergic transmission. Indeed, PDAs have a strong affinity for D_2_ receptors but activate them much less than dopamine, therefore they reduce signal transduction in case of excessive dopamine activation, as it happens in the mesolimbic pathway in schizophrenia (Stahl, 2001; Bandelow and Meier, 2003). At the same time, PDAs increase dopamine activation in hypodopaminergic brain circuits, such as the mesocortical pathway in schizophrenia (Lieberman, 2004). Thereby, PDAs may balance the levels of dopaminergic activity in the brain, improving both positive and negative symptoms. In particular, aripiprazole, brexpiprazole and cariprazine are characterized by D_2_ and 5HT_1A_ partial agonism. Despite their action on D_2_ results in a partial agonism, the binding affinity to this receptor is very strong. Brexpiprazole has a similar potency for 5HT_1A_, D_2_ and 5HT_2A_ binding. This molecule induces lower activation of D_2_ than aripiprazole, therefore it is closer to a pure antagonist (Stahl, 2016a,b, 2017). Cariprazine stands out for its additional very potent D_3_ partial agonist activity (Stahl, 2016b; Frankel and Schwartz, 2017). The D_3_ partial agonism appears to be linked to its potential therapeutic action on mood, cognition and reward/substance abuse, and, most importantly, to an improvement of negative symptoms in schizophrenia (Alptekin *et al.*, 2013; Corponi *et al.*, 2017; Rancans *et al.*, 2021).

### Neuroscience-based nomenclature

More recently, a new classification system for psychotropic medication named Neuroscience-based nomenclature (NbN) has been proposed (https://nbn2r.com). The NbN was created as an alternative to the indication-based nomenclature (which is based on the WHO’s Anatomical-Therapeutic-Chemical categorization system as established in 1976) (Zohar *et al.*, 2015; Sathyanarayana Rao and Andrade, 2016).

Traditionally, psychotropic medications have been classified by their main indication, such as antidepressants for treating depression or antipsychotics for treating psychosis. However, psychotropic drugs can be prescribed for several conditions that are not limited to their main or original indication. This mismatch can lead to patients doubting the effectiveness of a drug and the appropriateness of the prescription, which in turn has a negative impact on compliance. To overcome this issue and facilitate a rational prescription, NbN classified psychotropic medications based on their pharmacologic domain (i.e. the neurotransmitter system) and mechanism of action rather than their indication (Zohar and Kasper, 2016). The NbN classification for PDAs and FDAs are presented in Table 1.

**Table 1:** Neuroscience-based Nomenclature (NbN) classification of antipsychotic drugs (source: https://nbn2r.com/authors)

Former terminology (indication based)	Neuroscience-based nomenclature (pharmacological based)		Chemical names
	Pharmacology	Mode of action	
Antipsychotic (Neuroleptics, Major tranquillizers)	Drugs for psychosis		
Typical (first generation)	Dopamine	Receptor antagonist (D2)	Flupentixol, fluphenazine, haloperidol, perphenazine, sulpiride, trifluoperazine, zuclopenthixol
	Dopamine, serotonin	Receptor antagonist (D2, 5-HT2)	Chlorpromazine, thioridazine
Atypical (second generation)	Dopamine	Receptor antagonist (D2)	Amisulpride
	Dopamine, serotonin	Receptor antagonist (D2, 5-HT2)	Iloperidone, loxapine, lumateperone, lurasidone, olanzapine, ziprasidone, zotepine
	Dopamine, serotonin	Receptor partial agonist (D2, 5-HT1A) and antagonist (5-HT2)	Aripiprazole, cariprazine, brexpiprazole
	Dopamine, serotonin, noradrenaline	Receptor antagonist (D2, 5-HT2, NE alpha-2)	Asenapine, clozapine, risperidone, paliperidone
		Receptor antagonist (D2, 5-HT2) and reuptake inhibitor (NET)(metabolite)	Quetiapine

### Receptor binding affinity of antipsychotics

In preclinical trials, promising drug candidates are often screened with in-vitro techniques (e.g. ligand binding assay) to assess their receptor binding affinity. This step is essential to predict in-vivo efficacy of drug candidates (Hughes *et al.*, 2011; Koszła *et al.*, 2020). Indeed, in-vivo efficacy is closely related to receptor site occupancy. This parameter may be studied using molecular imaging techniques, such as positron emission tomography (PET) primarily (Cunha *et al.*, 2014). The use of PET in living humans or animals has become a standard tool to determine the interaction of molecules with target receptors (Varnäs *et al.*, 2013). In particular, the affinity of each drug quantifies the potency of interaction between the drug and a specific receptor, namely the concentration of drug required to occupy 50% of the same target receptor at equilibrium. This value corresponds to the equilibrium inhibitor constant (Ki). Lower Ki values equal to stronger binding (Correll, 2010a,b). Ki values for most common antipsychotic agents are shown in Table 2.

**Table 2:** Binding affinity of antipsychotics drugs at neurotransmitter receptors

Drug name	5-HT_1A_	5-HT_2A_	5-HT_2C_	5-HT_6_	5-HT_7_	α_1A_	α_2A_	α_2C_	D_2_	D_3_	H_1_	M_1_
Amisulpride	>10 000	8304	>10 000	4154	73.5	>10 000	1114	1540	2.2	2.4	>10 000	>10 000
Aripiprazole (PDA)	5.6	8.7	22.4	642.4	9.97	25.85	74.1	37.63	1.64	5.35	27.93	6778
Asenapine	2.5	0.06	0.03	0.25	0.13	1.2	1.2	1.2	1.3	0.42	1	8128
Brexpiprazole (PDA)	0.12	0.47	–	58	3.7	3.8	15	0.59	0.3	1.1	19	–
Chlorpromazine	2116	4.5	15.6	17	28.4	0.28	184	46	1.4	4.65	3.09	32.3
Cariprazine (PDA)	3	19	134	–	111	–	–	–	0.49	0.085	23	–
Clozapine	123.7	5.35	9.44	13.5	17.95	1.62	37	6	157	269.1	1.13	6.17
*cis*-Flupenthixol	8028	87.5 (HFC)	102.2 (RC)	–	–	–	–	–	0.35	1.75	0.86	–
Fluphenazine	1039.90	37.93	982.5	34.67	8	6.45	314.1	28.9	0.3	1.75	14.15	1095
Haloperidol	2066.83	56.81	4801	5133	377.6	12	801.5	403	0.7	3.96	1698	>10 000
Iloperidone	93.21	1.94	147	63.09	112	0.3	160	16.2	10.86	10.55	12	4898
Loxapine	2456	6.63	13.25	31	87.6	31	150.9	80	28.1	19.33	4.9	119.45
Lumateperone	–	0.5	173	–	–	73	–	–	32	–	>1000	–
Lurasidone	6.8	2	415	–	0.5	48	1.6	10.8	1.7	–	>10 000	>10 000
Olanzapine	2282	3.73	10.2	8.07	105.2	112	314	28.9	34.23	47	2.19	2.5
Paliperidone	616.6	0.71	48	2414	2.7	2.5	17.35	7.35	0.7	0.5	18.8	>10 000
Perphenazine	421	5.6	132	17	23	10	810.5	85.2	0.14	0.13	8	1500
Quetiapine	394.2	912	1843	948.75	307.6	22	3630	28.85	379	340	6.9	489
Risperidone	422.88	0.17	12	2057.17	6.6	5	16.5	1.3	3.57	2	20.05	>10 000
Thioridazine	144.35	27.67	53	57.05	99	3.15	134.15	74.9	2.2	1.5	16.5	12.8
Trifluoperazine	950	74	378	144	290.8	24	653.7	391.5	1.12	–	63	–
Ziprasidone	54.67	0.73	13	60.95	6.31	18	160	68	4.35	7.85	62.67	>10 000
Zotepine	470.5	2.7	3.2	6	12	7	208	106	25	6.4	3.21	18
Zuclopenthixol (cis-clopenthixol)	–	7.6	–	3	–	33	>4300	–	1.5	–	38	1700

Data are equilibrium inhibitor constant (Ki) values expressed in nM and derived from competition with radioligands on cloned human receptors. receptors unless otherwise specified. Lower Ki values correspond to higher affinities at each receptor.

5-HT1A, serotonin receptor; 5-HT2A, serotonin receptor type-2A; 5-HT2C, serotonin receptor type-2C; 5-HT6, serotonin receptor type-6; 5-HT7, serotonin receptor type-7; α1A, adrenergic α receptor type-1A; α2A, adrenergic α receptor type-2A; α2C, adrenergic α receptor type-2C; D2, dopamine receptor type-2; D3, dopamine receptor type-3; H1, histamine receptor type-1; M1, muscarinic acetylcholine receptor type-1; PDA, partial D2 agonist; HFC, human frontal cortex receptor; RB, rat brain receptor; RC, rat cloned receptor.

Based on binding affinity on D_2_, antipsychotics may be grouped in high (or strong) potency, medium potency and low potency. Low D_2_ affinity (having high Ki values) is linked with rapid dissociation from D_2_ (this is the case for example of clozapine and quetiapine), whereas compounds with high D_2_ affinity (showing very low Ki values) are displaced much more slowly from D_2_ (this is the case for example of haloperidol, fluphenazine and risperidone). Olanzapine has an intermediate dissociation time (Huhtala *et al.*, 2019; Roth *et al.*, 2000).

## Material and methods

### Search strategy, inclusion and exclusion criteria

A literature search was carried out, including articles published until September 2021. Databases used were *PubMed, Ovid* and *The Cochrane Central Register of Controlled Trials*. Both simple keywords and subject heading terms were used referring to the chemical names of FDAs (i.e. haloperidol, risperidone, paliperidone, ziprasidone, iloperidone, lurasidone, lumateperone, amisulpride, olanzapine, asenapine, zotepine, chlorpromazine, zuclopenthixol and fluphenazine), the chemical names of PDAs (i.e. aripiprazole, brexpiprazole and cariprazine) and terms referred to the schizophrenia spectrum (e.g. schizophrenia, schizoaffective disorder and schizophreniform disorder) or other psychotic disorders.

The relevance of abstracts was independently assessed by two reviewers (M.L. and G.F.), and the corresponding articles were classified as eligible, maybe eligible and not eligible. We evaluated the full text of eligible studies and those with unclear eligibility. Studies were included if they: (1) were unblinded/open-label or blinded clinical trials, case reports, case series, observational studies or animal studies; (2) focused either on patients diagnosed with any psychotic disorder or on animal models of psychosis; (3) reported a concurrent administration of a PDA (i.e. aripiprazole, brexpiprazole or cariprazine) and a strong FDA (i.e. ki = 0.14–4.35, namely haloperidol, risperidone, paliperidone, ziprasidone, lurasidone, amisulpride, asenapine, chlorpromazine, zuclopenthixol and fluphenazine) or olanzapine > 20 mg/day, a dose at which a D_2_ occupancy of ≥80% is expected for olanzapine (Kapur *et al.*, 1998); (4) were written in English and (5) described the clinical improvement or worsening associated with a combination of any PDA and strong FDA. We excluded studies if: (1) the concomitant use of FDA and PDA was limited to the switching phase; (2) PDAs were exclusively used for the management of antipsychotic-induced hyperprolactinemia or galactorrhea. A full-text reading and open discussion among the authors (M.L. and G.F.) were conducted to resolve doubts about studies with unclear eligibility. The bibliography of eligible articles was also screened for articles that may have been missed in the initial search.

### Data extraction and comparison of studies’ characteristics

For case reports and case series, the following data were extracted, when available: age, sex, diagnosis, time since disease onset, current antipsychotic medications, other concomitant medications, antipsychotic add-on, duration of observation until symptoms improvement/worsening, strategies used for managing clinical worsening or stabilize symptoms improvement. Unpaired Student’s *t*-tests were used to compare demographic and clinical variables, including the duration of observation, as well as PDAs and FDAs daily clinical equivalents in patients who showed clinical improvement and those showing clinical worsening after the combination of high-affinity FDA with PDAs.

## Results

**Fig. 1:**
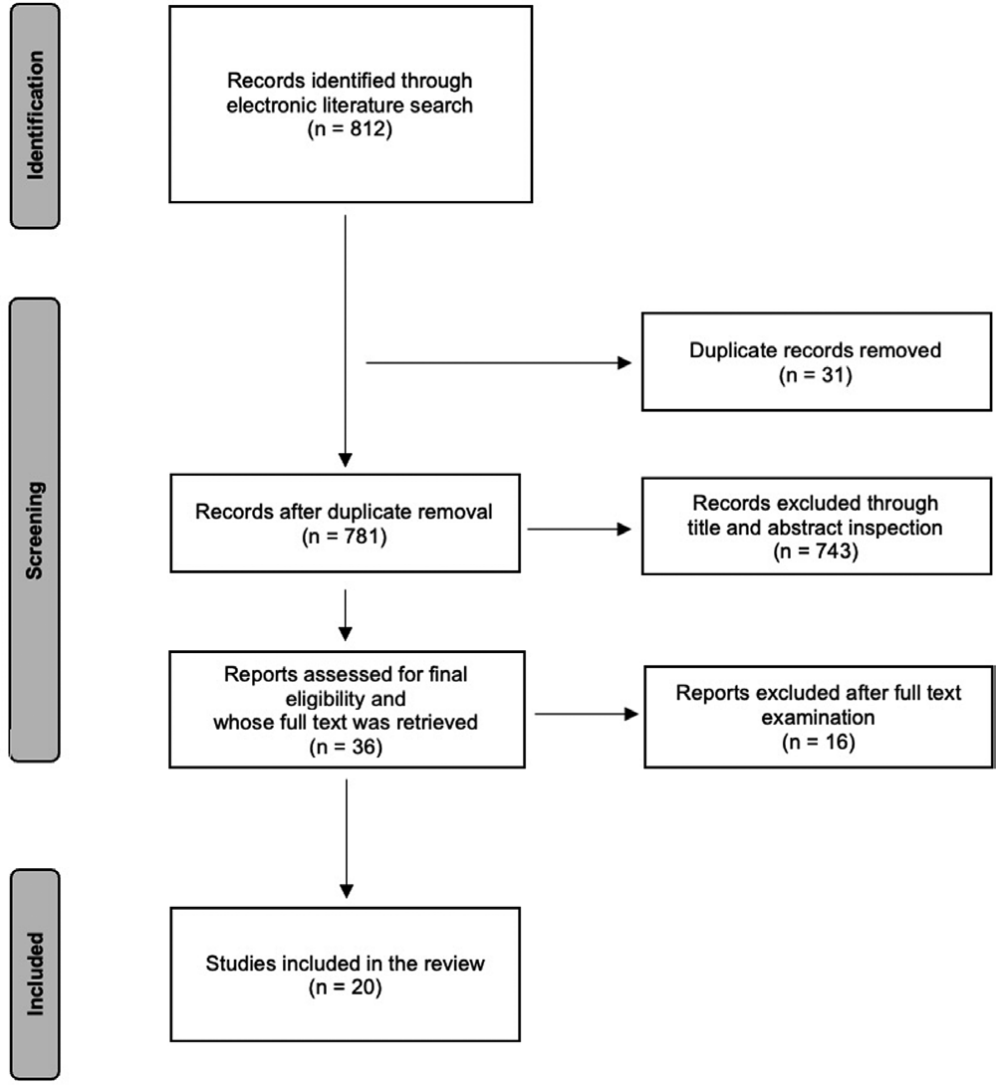
Flow diagram of the literature review.

The initial literature search returned a total of 812 records. Thirty-one studies were excluded because they were duplicates. After title and abstract screening, 743 studies were excluded. Thirty-six studies were assessed for final eligibility, and their full text was retrieved. In the end, 20 articles met the inclusion criteria and were therefore considered for qualitative synthesis (the study flow is depicted in Fig. 1).

The following types of studies were included:

(1) Twelve case reports (Burke and Lincoln, 2006; Grover, Sharan and Gupta, 2006; Ahuja and Lloyd, 2007; Kantrowitz *et al.*, 2007; Kapusta *et al.*, 2007; Kuo and Hwu, 2008; Leo *et al.*, 2008; Adan-Manes and Garcia-Parajua, 2009; Lecamwasam and Alexander, 2011; Huang and Chang, 2012; Noorani *et al.*, 2016; Evernden *et al.*, 2021);
(2) Five case series (DeQuardo, 2004; Ramaswamy *et al.*, 2004; Lea *et al.*, 2007; Raja, 2007; Li *et al.*, 2019);
(3) One double-blind, placebo-controlled RCT (Kane *et al.*, 2009);
(4) Two open-label clinical trials (Wang *et al.*, 2013; Gupta *et al.*, 2021);
(5) One animal study (Sonnenschein *et al.*, 2019).

No articles were found concerning the combined use of cariprazine or brexpiprazole with a strong FDA. All included articles reported the concomitant use of the PDA aripiprazole with a strong FDA. It should also be noted that aripiprazole was approved in 2002 in the United States and in 2004 in Europe. For this reason, all articles found were published from 2004 onwards.

### Case reports and case series characteristics

A total of 23 clinical cases were reported within the 12 case reports and five case series publications previously mentioned. Among these publications, 17 reported cases that did not support the combination treatment strategy (Table 3), and six reported in favor of it (Table 4). Two case reports suggested an improvement in tardive dyskinesia and excessive sweating following the addition of aripiprazole to previous therapy with strong FDAs (Kantrowitz *et al.*, 2007; Huang and Chang, 2012), while another case documented a worsening of akathisia (Lea *et al.*, 2007). In six other cases, aripiprazole was introduced on previous therapy with strong FDAs to improve side effects (hypothyroidism, extrapyramidal symptoms, sedation or iatrogenic weight gain); however, it was not possible to assess the effectiveness of this strategy in relieving the intended side effects due to the worsening of the psychopathological condition (5/6 cases) or the emergence of hyponatremia (1/6 cases).

**Table 3:** Cases of symptoms improvement after combination of partial D2 agonists with full, high affinity D2 antagonists in schizophrenia or schizoaffective disorder

Case number, study type	Reference	Sex, age	Diagnosis (disease onset)	Current AP (mg/day*)	Other concomitant medications (mg/day)	Reasons for add-on	Add-on AP (mg/day*)	Duration of observation (days)	Symptoms improved	Strategies to stabilize clinical improvement
1 Cr	(Kantrowitz *et al.*, 2007)	M, 53	SCZ (39 ya)	HALD 100 (every 30 days) + HAL 2	Lisinopril 10	Worsening tardive dyskinesia Mild hallucinations	ARI 10 (Stop HAL 2)	120	Improv. tardive dyskinesia Improv. hallucinations More socially	↑ARI 30 → complete resolution tardive dyskinesia and psychiatrically stable (after 17 months)
2 Cr	(Kuo and Hwu, 2008)	M, 41	SFD (<6 months ago)	ARI 15	-	Hallucinations (no complete remission)	HAL 2.5→ HAL 5	120	Stop hallucinations (complete remission)	↑ HAL 7.5 (stable remission of psychotic symptoms)
3 Cr	(Noorani *et al.*, 2016)	M, 29	SCZ (9 ya) MS	RSP 8	Sertraline 200 Biperiden 6	Still delusions and hallucinations	ARI 30		Complete remission	Maintained over time (36 months)
4 Cs	(Li *et al.*, 2019)	F, 35	SCZ/TRS (10 ya)	FLUXD 40 (every 2 weeks)		Partially relieved psychosis Delusion, hostility still severe	ARI LAI 400 (every 4 weeks)	90	Complete Remission	Stop FLUXD 40 (symptoms worsened) then FLUXD 20 (every 4 weeks) + ARI LAI 400 (every 4 weeks), (clinical stabilization)
5 Cr	(Huang and Chang, 2012)	F, 55	SCZ (25 ya) T2D	HAL15 Zotepine 350	Propranolol 60 Metformin 1,000 Biperiden 6 Glimepiride 2	Refractory hallucinations Excessive sweating	ARI 15 (↓ zotepine 200)	40	Hallucination improvement Stop sweating	↑ARI 30 Stop zotepine
6 Cr	(Evernden *et al.*, 2021)	F, 39	SCZ (4 ya)	ARI LAI 400 (every 4 weeks) ARI 10	Zopiclone 7.5	Worsening of psychosis	PAL 3 → PAL 9	23	Psychosis improvement	PAL LAI 100 (every 28 days) Stop oral PAL (12 months stable)

AP, antipsychotic; ARI, aripiprazole; F, female; FLUZD, fluphenazine decanoate; FLUXD, flupentixol decanoate; HAL, haloperidol; HALD, haloperidol decanoate; LAI, long acting injectable; M, male; MS, multiple sclerosis; PAL, paliperidone; RSP, risperidone; SCZ: schizophrenia; SFD: schizophreniform disorder; TRS: treatment-resistant schizophrenia, T2D: Type 2 diabete*s*, ya: years ago.

* Unless otherwise specified.

**Table 4:** Cases of symptoms worsening after combination of D2 partial agonists with full, high affinity D2 antagonists in schizophrenia or schizoaffective disorder

n. Type	Reference	Sex, Age	Diagnosis (disease onset	Current AP (mg/day*)	Other concomitant medications (mg/day)	Reasons for add-on	Add-on AP (mg/day*)	Duration of observation (days)	Symptoms worsened	Strategies for managing clinical worsening
1 Cs	(Ramaswamy *et al.*, 2004)	F, 43	SZA	ZPR 160 QTP 400	VPA 1500 Propranolol 30 Levothyroxine 0.05	QTP-related hypothyroidism	ARI 15 → ARI 30 (Taper QTP; switch VPA to CBZ)		Psychosis worsened	Trifluoperazine (as needed)
2 Cs	(Ramaswamy *et al.*, 2004)	F, 67	SZA (>35 ya)	ZPR 160	CBZ 200	Persisting psychotic symptoms	ARI 7.5 → ARI 30	60	Hallucinations worsened Insomnia Social isolation	
3 *Cs*	(Ramaswamy *et al.*, 2004)	M, 46	SCZ (chronic course)	RSP 3	VPA 1500	Persisting psychotic Symptoms	ARI 15	5	Delusions worsened	Stop ARI (improvement)
4 *Cs*	(DeQuardo, 2004)	M, 54	SCZ (>25 ya)	HALD 200 (every 4 weeks)	Benztropine (dosage not available)	Residual paranoid ideation Extrapyramidal symptoms	ARI 15	28	↑ Paranoid Ideation Aggressiveness	Stop ARI (improvement)
5 *Cs*	(DeQuardo, 2004)	M, 51	SCZ (>30 ya)	OZP 60		Persisting psychotic symptom	ARI 10	9	↑ Hallucinations worsened	Stop ARI (improvement)
6 *Cr*	(Burke and Lincoln, 2006)	M, 30	SCZ (5 ya)	ARI 10 0		Clinical stabilization	↑ARI 30		↑ Paranoid ideation Aggressiveness	↑HAL 20 (small improvement) →Stop ARI (improvement after 4 days)
7 Cr	(Grover *et al.*, 2006)	F, 24	SCZ	HAL 30		Persisting psychotic symptom	ARI 20	60	Psychosis worsened	↑HAL to 60 (no improvement) *→* Stop ARI (improvement)
8 Cr	(Ahuja and Lloyd, 2007)	F, 35	SZA	AMI 400 ARI 30	Lorazepam 1 (as needed)	Sedation Weight gain	ARI 10	9	↑Anxiety Hallucinations worsened ↑ Paranoid ideation	Stop ARI ↑ AMI 600 Add lorazepam 1
9 Cr	(Leo *et al.*, 2008)	F, 43	SCZ (12 ya)			Persisting psychotic symptom	HAL 5	7	↑QTc	Stop HAL (reduction QTc)
10 Cs	(Raja, 2007)	M, 30	SZA	AMI 500		Negative symptom	ARI 7.5		Functioning improved But: Delusions worsened Suicidal behavior Aggressiveness	¯ARI 5 (no improvement) *→* Stop ARI (improvement)
11 Cs	(Raja, 2007)	F, 59	SCZ (≥30 ya)	RSP 5		Negative symptom	ARI 7.5 (¯ RSP 4)	14	Hallucinations worsening Delusions worsened	¯ARI 2.5 (Psychosis worsened) →Stop ARI and ↑RSP 5 (improvement)
12 Cs	(Lea *et al.*, 2007)	M, 60	SCZ -	FLUZD 50 (every 2 weeks)	VPA 4500 Benztropine 3	Persisting psychotic symptom	ARI 10	21	Bizarre behavior Akathisia	Stop FLUZD and ↑ARI 15 (with ↑irritability, ↑agitation, aggressiveness) After 3 weeks: ↑ARI20 Restart FLUZD 75 (every 2 weeks) After 1 day: Stop ARI (with slow improvement)
13 Cr	(Lecamwasam and Alexander, 2011)	F, 56	SZA (chronic course)	FLUXD 40 (every 2 weeks) OZP	VPA 2000	Weight gain	ARI 30 (Stop OZP)		Hyponatremia	Stop ARI (normalization of sodium levels)
14 Cr	(Adan-Manes and Garcia-Parajua, 2009)	M, 23	SCZ (2 ya)	AMI 800	Biperiden 4	Better tolerance to previous monotherapy with ARI at the first-episode psychosis	ARI 10 →↑ ARI 20 (¯ AMI 400)	17	Hallucinations worsened Perplexity	¯ARI 10 and ↑AMI 800 (slowly improvement but still delusional telepathy); 5-day ARI withdrawal: improvement of delusional telepathy
15 Cr	(Adan-Manes and Garcia-Parajua, 2009)	Same patient	Same patient	AMI 800	Biperiden 4	Reintroduction of ARI (because of previous better tolerance) after being self-suspended for 5 days	ARI 10	5	Hallucinations worsened	Stop ARI (improvement)
16 Cr	(Kapusta *et al.*, 2007)	M, 37	SCZ (17 ya)	RSP 6		Improve negative symptoms	ARI 15	3	Hallucinations Delusions Aggressiveness Suicidal ideation	Stop ARI ↑ RSP 9 (improvement)
17 Cr	(Kapusta *et al.*, 2007)	Same patient	Same patient	RSP 9		Stepwise ARI reexposure to improve negative symptoms	ARI 2.5→ ARI 15	20	Hallucinations worsened	Stop ARI

AMI, Amisulpride; AP, antipsychotic; ARI, aripiprazole; CBZ, carbamazepine; CS, case series; CR, case report; FLUZD, fluphenazine decanoate; F, female; FLUXD, flupentixol decanoate; HAL, haloperidol; HALD, haloperidol decanoate; M, male; OZP, olanzapine, QTP, quetiapine; RSP, risperidone; SCZ, schizophrenia; SZA, schizoaffective disorder; VPA, valproic acid; ya: years ago; ZPR, ziprasidone.

* Unless otherwise specified.

The duration of clinical observation/follow-up during the combined treatment was significantly longer in cases showing clinical improvement than in cases with worsening of symptoms (78.6 days vs. 19.85 days; *t* = 3.979; *P* = 0.001; Table 5). No differences between the two groups were found in regard to age, sex and clinical equivalents of chlorpromazine for the used FDAs and aripiprazole (Table 5).

**Table 5:** Differences between patients showing improvements and patients showing symptoms worsening after combining high D2 affinity full dopamine antagonists and aripiprazole

	Cases showing symptoms improvement (*n* = 6)	Cases showing symptoms worsening (*n* = 17)	Test statistic	*P* value
Age, mean (SD)	42 (10.18)	42.24 (13.94)	*t* = 0.038	0.970
Sex, *n* males (%)	3 (50)	10 (58.82)	–	1^a^
Duration of observation, mean days (SD)	78.60 (45.11)	19.85 (19.28)	*t* = 3.979	0.001^b^
PDA daily dose^c^, mean (SD)	363.33 (149.76)	358.82 (176.10)	*t* = 0.056	0.956
FDA daily dose^c^, mean (SD)	495.17 (315.63)	615.06 (485.38)	*t* = 0.560	0.581

*t* = Student’s *t*-test statistic.

a Fisher’s exact test.

b P value<0.05.

c Chlorpromazine clinical equivalents [according (Gardner *et al.*, 2010)].

(1) Cases in favor of combining aripiprazole and a strong, full D_2_ antagonist

The mean age of patients was 42 years (ranging from 29 to 55 years old), and they were distributed equally by sex (three males and three females). Five patients were diagnosed with schizophrenia (including one with treatment-resistant schizophrenia) and one with schizophreniform disorder, but none with schizoaffective disorder (Table 3). Among these six patients, half had a disease duration of 10 years or more. In two out of six cases reported, the drug initially prescribed was aripiprazole [one of which was a Long-Acting Injectable (LAI) formulation], in two other cases haloperidol (one of which was a LAI formulation), in one case risperidone and in one case flupentixol decanoate (LAI formulation). In all cases, the reason for using a second add-on antipsychotic medication was incomplete remission (delusions and/or hallucinations were still present), while in two cases the intention was also to alleviate tardive dyskinesia and excessive sweating (Table 3).

(2) Cases against combining aripiprazole and a strong, full D_2_ antagonist

The mean age of the 17 cases was 42.24 years (ranging from 23 to 67 years old). They were almost equally distributed by sex (10 males and eight females). Twelve cases were diagnosed with schizophrenia and the remaining (n = 5) with schizoaffective disorder. Patients had a long-standing chronic disorder (>10 years) in most cases. In four cases, the initially prescribed antipsychotic was risperidone, in the other four cases amisulpride, in three cases haloperidol (one of which in LAI formulation), in two cases ziprasidone, in two cases aripiprazole, in one case fluphenazine decanoate (LAI formulation), in one case flupentixol decanoate (LAI formulation) and in the last case olanzapine 60 mg/day, which was three times the maximum recommended therapeutic dose. Of note, one patient was already taking a combination of aripiprazole and haloperidol as initial antipsychotic therapy, whereas two patients taking a combination of two antipsychotics having different D_2_ affinity (ziprasidone plus quetiapine and flupentixol decanoate plus olanzapine, respectively). The reasons for introducing aripiprazole included incomplete clinical remission (11 cases), side effects (five cases), or both (one case). In 14 of these cases, the combination therapy led to a worsening of psychotic symptoms. In one case, QTc prolongation was observed, and in another case, the patient developed hyponatremia. In all the cases there was a clinical improvement with the discontinuation of aripiprazole (Table 4).

### Clinical trials

Our literature search also identified one double-blind, placebo-controlled RCT lasting 16 weeks (Kane *et al.*, 2009). The sample consisted of patients with schizophrenia or schizoaffective disorder (*n* = 323), who had an incomplete response to a stable regimen of risperidone (4–8 mg/day) or quetiapine (400–800 mg/day) administered for at least 4* *weeks. Patients were randomly assigned to receive aripiprazole 2–15 mg/day (*n* = 168) or placebo (*n* = 155) as add-on. The primary endpoint was the medium change in the Positive and Negative Syndrome Scale (PANSS) total score. In addition, laboratory and vital parameters were measured and recorded, as well as any adverse event. This RCT indicated that the two groups (aripiprazole add-on and placebo add-on) were similar in both mean changes in the PANSS score and the incidence of adverse events. However, within the aripiprazole add-on group, a drop in prolactin and triglycerides levels were observed in the group taking risperidone (*n* = 63), but not in those taking quetiapine (*n* = 52). Although the aripiprazole add-on was generally well tolerated, the authors failed to prove that augmentation with aripiprazole 2– 15 mg/day can lead to a significant improvement in psychotic symptoms when compared to placebo. Nevertheless, the addition of aripiprazole in selected patients may be useful to decrease prolactin or triglycerides levels, although these observations need further replication (Kane *et al.*, 2009).

Two open-label trials were also identified (Wang *et al.*, 2013; Gupta *et al.*, 2021). Wang *et al.*(2013) conducted an 8-week study where aripiprazole (5– 20 mg/day) was added to risperidone (*n* = 19), amisulpride (*n* = 6), olanzapine (*n* = 12) or quetiapine (*n* = 6), in a total sample of 43 patients with a psychotic disorder (i.e. schizophrenia, schizoaffective disorder or bipolar disorder), who experienced any metabolic disturbance while taking one of the mentioned antipsychotics. The study outcomes were changes in weight/ body mass index, plasma levels of glycemic and lipidemic biomarkers, as well as PANSS scores. The mean dose of aripiprazole add-on was 9.9 ± 3.2 mg/day. Only patients treated with olanzapine had a significant reduction in body weight and triglyceride levels, while patients treated with either quetiapine or high D_2_ affinity FDAs (i.e. risperidone or amisulpride) did not show any metabolic improvement. Interestingly, aripiprazole augmentation led to improvements in PANSS scores and motor side effects in all patients, with no differences between antipsychotic groups (Wang *et al.*, 2013). Unfortunately, the discontinuation criteria included acute psychosis, and it is unknown how many patients exited the study due to psychopathologic worsening. In addition, the sample size for analyses in each individual antipsychotic group was limited, and patients treated with risperidone and amisulpride (strong FDAs) were merged with those treated with quetiapine in the analyses.

Gupta *et al.*, (2021) conducted another open-label trial over 12 weeks to investigate the impact of an adjunct fixed dose of aripiprazole (5 mg/day) on weight, waist circumference, glycemic and lipidemic biomarkers in patients with schizophrenia or schizoaffective disorder taking risperidone, olanzapine or clozapine (Gupta *et al.*, 2021). Fifty-five patients completed the study (18 were treated with olanzapine, 23 with risperidone and 14 with clozapine). Only the clozapine group showed significant weight loss and amelioration in laboratory parameters (total cholesterol, high-density lipoproteins, low-density lipoproteins, and triglycerides), while the olanzapine group achieved a significant reduction in triglyceride levels. In the risperidone group, none of the metabolic biomarkers or weight improved significantly. Furthermore, an improvement in functioning and symptom severity was shown for all patients, except the olanzapine group, which showed only an improvement in functioning but not in symptom severity. These findings, therefore, support the combination of a partial D_2_ agonist and a strong FDA such as risperidone with regard to a possible general psychopathologic improvement, although they do not suggest any advantage over possible metabolic side-effects (Gupta *et al.*, 2021).

### Animal studies

One relevant animal study was identified by our literature search (Sonnenschein *et al.*, 2019). This study investigated the impact of acute or repeated administration of aripiprazole in the methylazoxymethanol acetate (MAM) hyperdopaminergic rodent model, which shows anatomical, pathophysiologic (hippocampal dopaminergic hyperactivity) and behavioral abnormalities mirroring those observed in schizophrenia. The authors showed that administration of aripiprazole (1 mg/kg, intraperitoneal) in MAM rodents reversed the depolarization block state of dopaminergic neurons induced by acute administration of haloperidol (0.6 mg/kg, intraperitoneal) 1 h earlier. Five minutes after acute administration of aripiprazole, there was a significant increase in the number of active dopaminergic neurons (Sonnenschein *et al.*, 2019). This finding is compatible with the various reports of worsening psychotic symptoms in patients switching or adding aripiprazole to strong FDAs.

## Discussion

In this literature review, we summarized the available evidence on the possible effects of the combined use of PDAs and high D_2_ affinity, FDAs medications in patients with psychotic disorders or animal models of psychosis. Although the paucity of previous publications reporting on this combined treatment strategy does not allow us to draw firm conclusions (Takeuchi and Remington, 2013; Takeuchi *et al.*, 2018), we can still provide some information that may be useful in clinical practice.

From the data available, the combination of aripiprazole with a strong FDA does not appear to be helpful to increase the chances of response or to achieve a more rapid response compared to antipsychotic monotherapy. In this regard, a 16-week RCT showed no statistically significant change between PANSS total score at baseline and endpoint when aripiprazole add-on was compared to placebo add-on. The incidence of adverse events also remained similar between the two groups, showing no particular tolerability issues while suggesting a potential utility of aripiprazole add-on in reducing prolactin and triglyceride levels in the group taking risperidone plus aripiprazole (Kane *et al.*, 2009). The two retrieved open-label trials, lasting 8–12 weeks, suggested a potential psychopathologic improvement but no advantage on metabolic side effects resulting from the combination of aripiprazole and a strong FDA but these results appear less robust due to the very limited sample sizes, intrinsic limitations of the study design, and/or the pooling of FDAs without distinguishing those having high D_2_ affinity from others having low D_2_ affinity (Wang *et al.*, 2013; Gupta *et al.*, 2021).

Most of the case reports (17/23) suggested a potential risk of worsening psychotic symptoms during the combination therapy, although publication bias cannot be ruled out. The partial dopamine agonist properties of aripiprazole and its capacity to activate, albeit partially, the D_2_ signal transduction pathway are unlikely to explain the exacerbation of psychotic symptoms (Ahuja and Lloyd, 2008; Mahgoub, 2015). Because aripiprazole also has a high binding affinity for D_2_ (see Table 2), which appears to be even higher than risperidone and other strong FDAs, aripiprazole might displace strong FDAs from the D_2_ receptor site, reducing their antagonistic effects on D_2_ (and hence dopaminergic inhibition) (Roth *et al.*, 2000). This hypothesis is also supported by the reversal of the haloperidol-induced D_2_ blockade following acute administration of aripiprazole in a hyperdopaminergic rat model (Sonnenschein *et al.*, 2019). Of note, in 16 out of 17 reported cases of clinical worsening, aripiprazole was added (or, in one case, increased in dose) on a previous strong FDA treatment and not vice versa (see Table 4). In the one case of clinical worsening in which haloperidol was added to the ongoing aripiprazole therapy, no worsening of psychotic symptoms was reported but QTc prolongation (Leo *et al.*, 2008). On the other hand, two patients experienced an improvement in psychotic symptoms following the addition of a strong FDA on previous stable aripiprazole therapy (Kuo and Hwu, 2008; Evernden *et al.*, 2021). Therefore, adding aripiprazole on top of a strong FDA would appear to be a riskier strategy than adding a strong FDA on a previously ongoing aripiprazole therapy.

In addition, most of the clinical cases reported in the literature involved patients with a long duration of disease (>10 years). In this regard, a possible explanation for the observed clinical worsening could be that the chronic use of strong FDAs could cause a decrease in the activity of dopaminergic neurons, creating a functional hypodopaminergic state (Ahuja and Lloyd, 2007; Kapusta *et al.*, 2007). This hypodopaminergic state may result in an upregulation of postsynaptic dopamine receptors in the mesolimbic system (Silvestri *et al.*, 2000), which, when combined with the high D_2_ binding affinity of PDAs, may cause aripiprazole to act similarly to a full postsynaptic D_2_ agonist and thus worsen psychotic symptoms. A similar mechanism has also been described in the so-called dopamine supersensitivity psychosis, in which a relapse of psychosis may occur after switching to a PDA from a strong FDA (Takase *et al.*, 2015; Chouinard *et al.*, 2017; Kanahara *et al.*, 2020; Russell *et al.*, 2022).

Interestingly, among the 17 cases that showed clinical worsening after initiation of aripiprazole plus a strong FDA, five had a prior diagnosis of schizoaffective disorder. This same diagnosis was never found in reports showing clinical improvement, although this finding is likely due to chance. We also observed that clinical improvement required about 11 weeks, while clinical worsening, when it was reported, occurred after about 3 weeks from the beginning of the combination therapy. However, the mere examination of the clinical cases documented in the literature does not allow to draw conclusions on the possible clinical predictors of clinical worsening or improvement following combined treatment with PDAs and strong FDAs.

Our literature review was limited by the absence of data on the combined use of a strong FDA and the PDAs cariprazine or brexpiprazole. In addition, the absence of well-powered clinical trials with larger sample sizes that clearly distinguish high from low D_2_ affinity FDAs prevents us from drawing solid conclusions. Therefore, the clinical interpretation that we derived should be considered with caution.

### Conclusion

Overall, this literature review suggests that the combination of the partial D_2_ agonist aripiprazole and a strong, full D_2_ antagonist does not help to achieve a more rapid response compared to maintaining antipsychotic monotherapy in psychotic disorders. In this regard, we observed that clinical improvement required about 11 weeks, while clinical worsening, when it was reported, occurred after about 3 weeks from the beginning of the combination therapy. Thus, the first few weeks of the combination require careful monitoring because of the possible emergence of symptom worsening, especially if aripiprazole is added on top of previous stable therapy with a strong FDA, and the patient has a longer duration of disease. Achieving clinical improvements during combination therapy with aripiprazole and a strong FDA may take longer on average. Adding aripiprazole on a strong FDA is likely to be a riskier strategy than adding a strong FDA on an already ongoing aripiprazole monotherapy in terms of the risk of worsening psychotic symptoms.

Studies on short- and long-term effects are required to examine the safety and tolerability of cariprazine or brexpiprazole combination with a strong FDA. Well-powered, placebo-controlled, randomized trials are also needed to investigate the usefulness of combining a PDA and strong FDAs and derive more solid clinical recommendations.

## Acknowledgements

### Conflicts of interest

A.S. is or has been a consultant/speaker for Abbott, Abbvie, Angelini, AstraZeneca, Clinical Data, Boehringer, Bristol-Myers Squibb, Eli Lilly, GlaxoSmithKline, Innovapharma, Italfarmaco, Janssen, Lundbeck, Naurex, Pfizer, Polifarma, Sanofi, Servier, and Taliaz. C.F. was a speaker for Janssen. For the remaining authors, there are no conflicts of interest.
